# Embracing model-based designs for dose-finding trials

**DOI:** 10.1038/bjc.2017.186

**Published:** 2017-06-29

**Authors:** Sharon B Love, Sarah Brown, Christopher J Weir, Chris Harbron, Christina Yap, Birgit Gaschler-Markefski, James Matcham, Louise Caffrey, Christopher McKevitt, Sally Clive, Charlie Craddock, James Spicer, Victoria Cornelius

**Affiliations:** 1 Oxford Clinical Trials Research Unit, Centre for Statistics in Medicine, NDORMS, University of Oxford, Botnar Research Centre, Windmill Road, Oxford OX3 7LD, UK; 2 Clinical Trials Research Unit, Leeds Institute of Clinical Trials Research, University of Leeds, Leeds LS2 9JT, UK; 3 Edinburgh Clinical Trials Unit, Usher Institute of Population Health Sciences and Informatics, University of Edinburgh Medical School, Teviot Place, Edinburgh EH8 9AG, UK; 4 Roche Pharmaceuticals, 6 Falcon Way, Shire Park, Welwyn Garden City AL7 1TW, UK; 5 Cancer Research UK Clinical Trials Unit, Institute of Cancer and Genomic Sciences, College of Medical and Dental Sciences, University of Birmingham, Birmingham B15 2TT, UK; 6 Boehringer Ingelheim Pharma GmbH & Co. KG, Biostatistics and Data Sciences, Birkendorfer Strasse 65, Biberach an der Riss 88400, Germany; 7 AstraZeneca, DaVinci Building, Melbourn Science Park, Royston SG8 6HB, UK; 8 School of Social Work and Social Policy, Trinity College Dublin, College Green, Dublin 2, Ireland; 9 Division of Health and Social Care Research, Faculty of Life Sciences and Medicine, King’s College London, London, UK; 10 Edinburgh Cancer Centre, Western General Hospital, Edinburgh EX4 2XU, UK; 11 Centre for Clinical Haematology, Haematology – University Hospitals Birmingham NHS Foundation Trust, Queen Elizabeth Hospital, Queen Elizabeth Medical Centre, Birmingham B15 2TH, UK; 12 Division of Cancer Studies, Bermondsey Wing, Guy’s Hospital, Great Maze Pond, London SE1 9RT, UK; 13 Imperial Clinical Trials Unit, Imperial College London, Stadium House, 68 Wood Lane, London W12 7RH, UK

**Keywords:** model-based design, dose-finding trials, phase I, CRM, 3+3

## Abstract

**Background:**

Dose-finding trials are essential to drug development as they establish recommended doses for later-phase testing. We aim to motivate wider use of model-based designs for dose finding, such as the continual reassessment method (CRM).

**Methods:**

We carried out a literature review of dose-finding designs and conducted a survey to identify perceived barriers to their implementation.

**Results:**

We describe the benefits of model-based designs (flexibility, superior operating characteristics, extended scope), their current uptake, and existing resources. The most prominent barriers to implementation of a model-based design were lack of suitable training, chief investigators’ preference for algorithm-based designs (e.g., 3+3), and limited resources for study design before funding. We use a real-world example to illustrate how these barriers can be overcome.

**Conclusions:**

There is overwhelming evidence for the benefits of CRM. Many leading pharmaceutical companies routinely implement model-based designs. Our analysis identified barriers for academic statisticians and clinical academics in mirroring the progress industry has made in trial design. Unified support from funders, regulators, and journal editors could result in more accurate doses for later-phase testing, and increase the efficiency and success of clinical drug development. We give recommendations for increasing the uptake of model-based designs for dose-finding trials in academia.

Dose-finding trials are essential in drug development as they establish a recommended dose for later-phase testing. We need reliable, efficient phase I trial designs for faster, cheaper drug development.

Phase I trials aim to find a recommended dose based on a target/acceptable toxicity level or some other criteria and use algorithm- or model-based designs (Braun, 2014). We focus here on trials determining the maximum-tolerated dose (MTD), which is the highest dose of drug or treatment that does not cause too many patients unacceptable side effects. Algorithm-based designs, such as the 3+3 (Carter, 1987), use rules fixed during trial design to select the MTD and allocate patients to a dose level. Dose levels are assigned using information from patients at one dose level. Model-based designs, such as continual reassessment (CRM) (O'Quigley *et al*, 1990), allocate patients to a dose level using a targeted toxicity rate and a statistical model describing the dose–toxicity relationship between the dose levels. When a new patient is registered to the trial, the model is updated using all available information on all registered patients and the dose for the new patient is agreed using the model-suggested dose as a guideline. Information from every patient at every dose level is used to decide the next dose. The model recommends the final MTD at trial completion.

Although statisticians recommend model-based designs, most phase I trials use algorithm-based designs (Rogatko *et al*, 2007; van Brummelen *et al*, 2016). We need to understand why statisticians’ endorsement of model-based designs is often ignored (Jaki, 2013; Paoletti *et al*, 2015) so that we can act appropriately to improve uptake.

We summarise the benefits of model-based designs and statisticians’ opinions of why these designs are neglected from the literature. We survey researchers’ reasons for avoiding these designs. We demonstrate how to overcome these barriers using a real-world example and provide recommendations and solutions to remove perceived barriers to using model-based designs.

## Materials and methods

### Literature review

We conducted a literature review, searching PubMed on 13 May 2015 and Embase on 8 June 2015 for ‘3+3’, ‘CRM’, and general terms. Supplementary Tables A and B show our search strategies.

### Survey

We identified four themes in studies examining uptake of adaptive designs and Bayesian methods (Chevret, 2012; Jaki, 2013; Morgan *et al*, 2014; Dimairo *et al*, 2015a): resources, knowledge, training, and implementation. We developed survey questions (Supplementary Table C) to identify barriers within these themes. We included one question for statisticians on software and another for other respondents on statistical support.

The survey was sent to clinical academics working with AstraZeneca, chief investigators (CIs) involved in trials reviewed and approved by the Cancer Research UK (CRUK) New Agents Committee (NAC), and International Clinical Trials Methodology Conference delegates (ICTMC 2015) who registered for a dose-finding studies workshop.

The frequency and proportion of each response was calculated for each questionnaire item. The proportion of respondents who considered each item a barrier was calculated by combining the numbers who rated the item ‘always’ and ‘often’ or ‘strongly agree’ and ‘agree’.

## Results

### Model-based approaches today

Model-based approaches have been neglected since their introduction in the 1990s. They were used in 1.6% of phase I trials published 1991–2006 (Rogatko *et al*, 2007), increasing to only 6.4% by 2012–2014 (van Brummelen *et al*, 2016).

### Benefits of model-based approaches

Model-based designs for phase I trials offer flexibility, superior operating characteristics, and scope for extension (Le Tourneau *et al*, 2009).

#### Flexibility

Model-based approaches allow complete flexibility in defining a target dose-limiting toxicity rate and enable the MTD to be estimated with the required degree of precision. The MTD may therefore be defined as the highest dose with a dose-limiting toxicity rate below the target threshold, with the threshold chosen based on the trial patient population and prior knowledge of the evaluated drug. Model-based designs can accommodate different underlying dose–response curve shapes. Doses can be skipped to accelerate escalation or de-escalation, and new dose levels can be defined during the trial. The risk of dose-limiting toxicity events in later treatment cycles can also be evaluated (Paoletti *et al*, 2015).

#### Superior operating characteristics

Across different dose–toxicity curves, model-based designs select the dose with the target dose-limiting toxicity rate more often than 3+3 designs (Thall and Lee, 2003; Boonstra *et al*, 2015) and expose fewer patients to doses with dose-limiting toxicity rates above or below the target level during the trial (Iasonos *et al*, 2008; Le Tourneau *et al*, 2012). The safety of model-based designs is evaluated at the design stage using simulation, with incorporation of overdose control where appropriate, and checking that decisions are sensible. Simulations have shown that more patients are likely to be overdosed or treated at subtherapeutic doses with 3+3 designs than model-based designs (Babb *et al*, 1998). Model-based designs also outperform 3+3 designs when attribution errors for adverse events occur (Iasonos *et al*, 2012). Unlike 3+3 designs, model-based designs can accommodate many candidate doses without substantially affecting the designs operating characteristics (Jaki *et al*, 2013). A CRM design achieved a recommended MTD after a median of three to four fewer patients than a 3+3 design (Onar *et al*, 2009).

#### Extended scope

Model-based approaches can be varied to suit a particular intervention and trial. For example, they can incorporate toxicity grade information (Iasonos *et al*, 2010; Doussau *et al*, 2015), combination treatments (Mandrekar *et al*, 2007), non-binary end points such as biomarker, pharmacokinetic or pharmacodynamics measures (Calvert and Plummer, 2008), time-to-event information (Cheung and Chappell, 2000), and multiple treatment schedules (O’Quigley and Conaway, 2011).

With so much evidence supporting model-based designs, why do trial teams avoid them?

### Possible barriers to model-based approaches

The literature offers many opinions, and little empirical evidence, on why model-based designs are neglected.

Algorithm-based designs such as the 3+3 design are the most used oncology dose-escalation design and therefore oncologists are exposed to and become familiar with it, and the literature offers many practical examples (Rogatko *et al*, 2007). Clinicians using 3+3 designs often informally incorporate available data from lower doses and use their experience of previous trials when deciding dose allocations. Many believe that 3+3 designs are flexible, practical, functioning phase I designs (Ishizuka and Ohashi, 2001).

Model-based designs are seen as a ‘black box’ approach to dose escalation that makes clinical interpretation of model parameters difficult during design development. Statistical analysis is needed after each dose cohort, which appears time consuming and complicated. Despite strong counterevidence (O’Quigley, 1999), many believe that model-based designs are less efficient than 3+3 designs in terms of time-to-complete and numbers treated above the MTD (Korn *et al*, 1994). Our experience is that clinicians also worry that they cannot overrule a model’s dose-escalation recommendations, and often cite 3+3 designs as providing safe, conservative estimates of the MTD.

Clinicians may find model-based designs’ need for prior information counterintuitive. As a phase I trial’s start is rife with uncertainty, many erroneously believe that the model’s required information can only be acquired after the trial starts. The reliability of dose-escalation decision-making is thought to be heavily dependent on weak prior assumptions.

Relative to algorithm-based designs, there are few published practical examples of model-based designs; although the systematic review of Iasonos and O'Quigley (2014) provides references of CRM published trials from 2001 to 2013, Levy *et al* (2006) and Paoletti *et al* (2006) are useful descriptions of trials, and Iasonos *et al* (2015) guides protocol writing.

Setting up model-based designs requires time and expertise. The statistician and CI must interact frequently, requiring access to a statistician and time for design development (Morita *et al*, 2007; Jaki, 2013; Dimairo *et al*, 2015b). Even when statistical advice is available, choosing the most appropriate design for a particular trial from the many designs on offer is challenging. Add time constraints due to funding application deadlines, and it is unsurprising that clinicians prefer ‘simple’, familiar methods (Jaki, 2013; Dimairo *et al*, 2015b).

### Survey results: perceived barriers

We surveyed clinicians, statisticians, researchers, and trial managers to ascertain which of the barriers identified in our literature review are currently affecting the medical research community. We received responses from 14 of the 62 (23%) clinical academics working with AstraZeneca, 22 of the 45 (49%) CIs involved in trials reviewed and approved by the CRUK NAC, and 43 of the 93 (46%) participants registered for the ICTMC 2015 workshop giving an overall response rate of 40% (79 out of 200).

Table 1 summarises the survey participants’ disciplines and experience. The majority were CIs (40%) or statisticians (39%), representing a range of experience levels. Around half had used non-algorithm-based methods. Of the 30 participating statisticians, 53% reported access to specialised statistical software to support design and analysis of model-based approaches. Of the 30 participating CIs, 83% reported access to statistical support to undertake a non-rule-based design.

When designing a new trial, 53% of the respondents said they always or often considered an alternative to algorithm-based methods. However, 16% reported a poor experience using alternative designs where reasons given included the reliance of real-time data entry for CRM, which slowed down decision making and less data available on other doses to model the efficacy curve and undertake biomarker exploratory analysis (Supplementary Table D).

**Figure 1:**
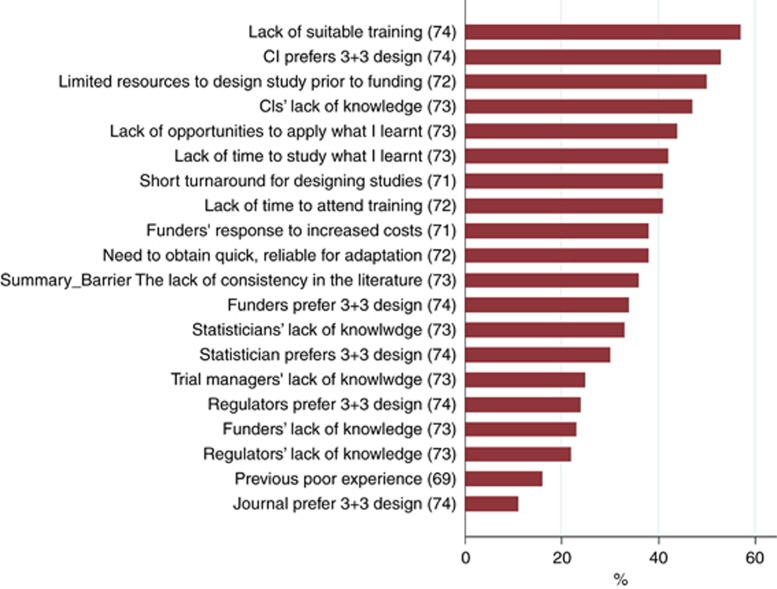
**Percentage of respondents identifying each item as a barrier to implementing model-based designs.** See Supplementary Table C for all items. CI=chief investigator.

Figure 1 shows the proportion of respondents who identified each questionnaire item as a barrier to implementing model-based designs. The top three barriers were lack of training to use alternative approaches to algorithm-based designs (57%), CIs’ preference for 3+3 designs (53%), and limited resources for study design before funding (50%). Many other items were also rated as barriers by a large proportion of the respondents, such as lack of opportunities to apply learnt skills in using alternative approaches to algorithm-based designs and how quickly studies must be designed.

We collected free-text comments to capture other attitudes or barriers (Supplementary Table D). The most common theme was respondents’ experience of a model-based study that was slower or larger than a typical 3+3 design. Other concerns about model-based designs themes were: difficulties of real-time data capture, limited data on alternative doses for pharmacodynamics, lack of experienced statisticians, and not selecting a safe dose. Improving uptake themes were: ‘selling’ model-based approaches to CIs and funders, accessible software, and consensus on which model-based approach to use.

### Resources to support model-based design

Resources exist to help trial designers overcome some of the identified barriers.

**Software** UK-based non-industry statisticians have access to free CRM software programmes, such as crmPack, dfcrm, and bcrm (http://cran.r-project.org), R shiny apps Web Application for simulating operating characteristics of the Bayesian CRM (Wages, 2017), and EWOC (Cedars-Sinai, 2017).

#### Working groups

The Medical Research Council (MRC) funds a National Network of Hubs for Trial Methodology Research to undertake trials methodology research. The Network’s adaptive design working group promotes methodology and supports using innovative designs through workshop-based advice forums, tutorial papers, individualised support, and software development (MRC Hubs for Trials Methodology Research, 2015). The National Institute of Health Research Statistics Group supports translating statistical methods into practice. Its early-phase clinical trial initiative focuses on dose-finding studies and works with academic researchers and industry collaborators (NIHR Statistics Group, 2016).

#### Guidelines

As the statistical language used in CRM studies can inhibit understanding, published guidelines indicate which operating characteristics to summarise to help the entire medical, scientific, and statistical team evaluate a proposed design (Iasonos *et al*, 2015). Other guidelines focus on protocols (Petroni *et al*, 2017).

#### Learning from industry

Many pharmaceutical companies have overcome practical barriers to implementing model-based designs, motivated by inaccurate doses from 3+3 trials causing failed phase II and III studies. Academia can use these companies’ experiences (Table 2).

#### Practical examples

There are few published practical examples of model-based designs (van Meter *et al*, 2012) or suggested design modifications (Potter, 2002; de Lima *et al*, 2010; Jones *et al*, 2012; Wages *et al*, 2015) to help overcome perceived barriers. In Box 1, we present our experiences developing a CRM-based phase I trial to show an example of the process.

## Discussion

Despite the vast literature outlining the statistical benefits of model-based designs over algorithmic designs, model-based methods are rarely used. In addition, in recent years there have been advances in therapeutic strategies where the relationship between dose and toxicity is less obvious and for which model-based designs are necessary. The literature suggests that time constraints, limited statistical resources, and few published practical examples are the greatest barriers to implementation, and that resources, knowledge, and training are the key to improving uptake.

We surveyed clinicians, statisticians, and trialists interested in dose-finding trials on their opinions of what stops trial teams from using model-based designs. We approached 200 people; 40% responded. As we targeted a convenient sample of researchers involved or interested in phase I methodology, our results may not be representative of the dose-finding research community. Those acquainted with model-based designs may have been more likely to respond, as 83% of the clinical respondents reported access to statistical support to implement a model-based approach, and 53% of the statistician respondents reported access to suitable software. Although the sample may not be a cross-section of the dose-finding community, the opinions of experienced researchers familiar with model-based methods provide valuable insights. Our results agree with previous surveys of similar scope but wider focus (Jaki, 2013; Dimairo *et al*, 2015b).

**Figure 2 fig2:**
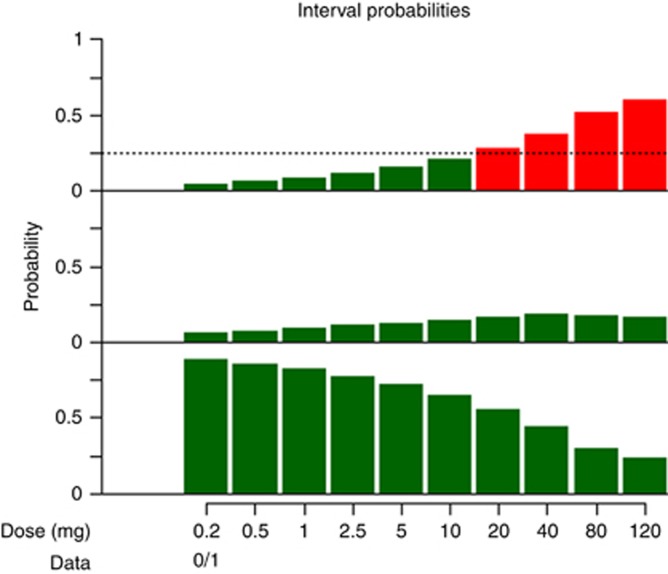
**Toxicity interval probabilities for all prespecified dose levels after one patient has been treated with 5 mg, showing the probabilities for (top) over-, (middle) target, and (bottom) undertoxicity.** With green indicating safe doses and red indicating unsafe doses, this shows the current dose decision can be based solely on overtoxicity since only the overtoxicity graph has red doses. We wish to increase the dose if we can; the current patient took 5 mg, but 10 mg would also be safe; thus, the model proposes 10 mg for the next patient.

We did not identify one obvious barrier. Many barriers were considered important by a large proportion of respondents, including clinician and statistician lack of knowledge; clinician, statistician, and funder preferences; lack of training and time for study design before funding; and funder responses to increased costs. A step change in practice will require a multifold approach targeting funders, clinicians, and statisticians.[Fig fig2][Table tbl3][Table tbl4]

We discuss the identified barriers to uptake, the progress thus far, and suggestions for facilitating change, referring to our real-world example in Box 1 (see summary in Table 4).

### Expectations

Many of our respondents avoided model-based designs as previous attempts had resulted in larger or slower trials than expected. Model-based designs do not necessarily mean smaller phase I trials. Instead, these designs more accurately identify the correct dose for future studies, reducing dose re-evaluations and improving efficiency and success in the more expensive later stages of drug development.

### Training

Our respondents rated lack of training as the greatest barrier to using model-based designs. The MRC’s adaptive design working group (MRC Hubs for Trials Methodology Research, 2015) promotes the use of model-based design through publications, workshops, expert advice forums, and individualised support for statisticians. There are some explanatory papers (Garrett-Mayer, 2006). However, little practical training exists on designing and implementing CRM-based designs throughout a trial’s life. More publications on the practicalities are required.

### Lack of time

Two frequently reported barriers were how quickly studies are designed and lack of time to study and apply methods. Promoting earlier, frequent discussion of trial ideas between clinicians and statisticians may mitigate these time constraints. Our real-world example shows that ongoing discussion between statisticians and clinicians helps ensure that the final design reflects clinical opinion.

### Design evaluation

Our survey highlighted lack of resources for evaluating trial designs as a barrier. The example in Box 1 shows that software templates can speed up design evaluation. Sufficient software training and support during grant development would be very valuable.

### Regulators

Over 20% of our respondents believed that regulators prefer 3+3 designs and a similar percentage felt regulators lack knowledge of other designs. However, UK regulators do endorse other trial designs, and the European regulatory guidance on first-in-man trials (Committee for Medicinal Products for Human Use, 2007) does not dictate a design (ICH E4, 1994; Hemmings, 2006; European Medicines Agency, 2014; Europeans Medicines Agency, 2015). Experiences from pharmaceutical companies show that model-based designs are readily accepted by health authorities and ethics boards.

A model-based phase I trial design must be described and justified in a clinical trial authorisation application like any other design choice. Regulators evaluate the appropriateness of the chosen method in the application’s context. We encourage regulators to make their position clear to clinicians and statisticians.

### Funders

Funders drive the academic clinical research agenda by setting strategic health priorities and commissioning research projects. They influence the direction and quality of research, as researchers aim to deliver what funders demand. Funders can play a pivotal role in encouraging better statistical methods in the design and analysis of dose-finding studies by setting strategic objectives, implementing rigorous statistical peer review, and integrating statistical expertise into their processes. We encourage funding bodies and ethics committees to question the use of algorithm-based designs, conduct statistical reviews of all phase I trial applications, and embrace model-based studies.

Ignorance of the benefits of model-based designs and disadvantages of algorithm-based designs is blocking wider implementation of more efficient phase I trial designs. Educating funding bodies, ethics committees, and regulatory agencies via tailored training sessions will enable more scientific appraisal of phase I trial designs. This will provide a greater return on investment: studies will produce more reliable results, increasing the likelihood of successful drug development. We can extend these principles to publications. Journal editors and reviewers should question study designs and how they affect the reliability of dose recommendations for future studies.

## Conclusion

By encouraging earlier clinical and statistical discussion, highlighting available training resources and practical examples, and calling for education for funders and other review committees, we hope to help overcome the barriers to model-based designs identified here. Implementing model-based designs will generate more accurate dose recommendations for later-stage testing and increase the efficiency and likelihood of successful drug development.

## Figures and Tables

**Table 1:** Characteristics of the survey participants

**Question**	**Response**	***N***	**%**
**Are you (*N*=79)**			
	Chief investigator	31	39
	Funder	1	1
	Other	14	18
	Statistician	30	38
	Trial manager	3	4
**How long have you worked with dose-finding studies? (years) (*N*=78)**			
	0–2	10	13
	3–5	12	15
	6–10	12	15
	11–12	14	18
	20+	5	6
	New to topic	25	32
**Have you ever been involved in a dose-finding study that, rather than using 3+3 or another rule-based design, used an alternative? (*N*=76)**			
	No	37	49
	Yes	37	49
	Don't know	2	3
**Do you have access to software to support alternative approaches to 3+3 and other rule-based designs? Statisticians only (*N*=30)**			
	Don't know	6	20
	No	8	27
	Yes	16	53
**Is appropriate statistical support available to you to undertake alternative approaches to 3+3 and other rule-based designs? Chief investigators only (*N*=30)**			
	Don't know	2	7
	No	3	10
	Yes	25	83
**When designing a trial, how often do you consider alternatives to 3+3 and rule-based designs (*N*=77)**			
	Always	22	29
	Often	19	25
	Not very often	15	19
	Never	4	5
	Don't know	17	22

**Table 2:** Pharmaceutical companies’ steps to promote model-based designs^a^

**Company**	**Model-based designs**	**Key activities**
AstraZeneca	All early oncology dose-escalation trials since 2014	Education programme
		Routine trial simulation software
		Standard method for prior toxicity–response curves
		All possible dose–response scenarios prepared for dose-escalation meetings
Roche Pharmaceutical Research and Early Development	Standard approach for oncology dose-escalation studies	Developed R software package, crmPack, for simulating, visualising, and running CRM studies
		Joint scientific forums between statistical and medical colleagues
		Examples of deployed designs
Boehringer Ingelheim	Standard approach for dose-finding (two-parameter Bayesian logistic regression model (Neuenschwander *et al*, 2008) with overdose control)	Expert statistics group provides support
		Training for statisticians and non-statisticians
		Template text for clinical trial protocols
		Template R and SAS (SAS Institute Inc., Cary, NC, USA) programmes for protocols, steering committee meetings, and clinical trial reports

Abbreviation: CRM=continual reassessment method.

a Information provided by authors of this paper.

**Table 3 tbl3:** Toxicity interval probabilities for all prespecified dose levels after one patient has been treated with 5 mg

	**Probability of toxicity rate in**			
**Dose (mg)**	**(0–0.16) Undertoxicity**	**(0.16–0.33) Target toxicity**	**(0.33–1) Overtoxicity**	**Mean toxicity rate**
0.2	0.889	0.065	**0.046**	0.056
0.5	0.860	0.075	**0.065**	0.071
1	0.828	0.089	**0.083**	0.086
2.5	0.770	0.115	**0.115**	0.112
5	0.717	0.128	**0.155**	0.140
10	0.649	0.144	**0.207**	0.177
20	0.559	0.163	*0.278*	0.228
40	0.439	0.186	*0.374*	0.300
80	0.301	0.179	*0.520*	0.403
120	0.231	0.167	*0.601*	0.473

Numbers in bold indicate safe, and those in italics indicate unsafe.

**Table 4 tbl4:** Summary of recommendations to increase the uptake of model-based designs in academia

	**Item**	**Recommendations**
Misconceptions	CI’s disillusioned with the idea that model-based ideas are more efficient	Address perceptions of ‘efficiency’ for model-based designs. Communicate that this means more often accurately identifying the correct dose rather than meaning an individual study will be shorter in duration or have a lower sample size
	Perception that regulators prefer 3+3	Communicate that UK regulators do endorse other trial designs and European regulatory guidance does not dictate use of a particular trial design
Training	Supporting uptake of model-based designs by statisticians and CIs	While training courses for utilising bespoke expensive software exist, training courses providing a broad academic introduction to the field and utilising free or inexpensive software need to be developed
		More publications on the practicalities of setting up and running model-based trials
	Appraisal of studies by funding bodies and ethics committees	Develop tailored training sessions for key partners to support a thorough scientific appraisal of proposed designs of phase I trials
	Model-based dose-finding experienced statisticians contact	Develop a forum for contacting experienced statisticians
Design and evaluation	Lack of time to design and evaluate a model-based approach	Promote the need for early discussions between CI and statisticians to allow time to develop and evaluate
		Develop software and protocol templates
Funding	Question routine use of 3+3 designs	Encourage funders to question the use of algorithm-based designs and embrace the idea of more efficient model-based studies
	Lack of statistical review for applications	Include statistical representation on funding board

Abbreviation: CI=chief investigator.

## Box 1. Planning and executing a phase I trial with a Bayesian design: real-world example

Objective: Perform an open-label single-compound dose-escalation trial to find a new compound’s MTD.

The team agreed to consider a model-based approach to explore doses ranging from 0.2 to 120 mg. The statisticians explained the proposed design to the clinicians. They emphasised the advantages of a model-based design over a 3+3 design: flexibility in choosing the next dose and in cohort sizes, and superior operating characteristics. As reliable results were important, the medical trial team agreed to use a Bayesian model-based approach.

The model-based design needed a prior probability distribution capturing the clinicians’ prior beliefs about toxicity at different doses and their uncertainty in these beliefs. The statisticians and clinicians discussed their expectations of the new compound’s toxicity. As no clinical data were available, the clinicians estimated the toxicity rates at specific doses for best-case, worst-case, and expected scenarios. As these estimates were based on scant information, they were used to form a minimally informative mixture prior; the information had a small effect on the trial.

The statisticians calculated the prior effective sample size to show the clinicians that the prior’s information would not overrule information gathered in the trial. The study design included safety constraints, such as no dose skipping and not recommending doses when the probability of a dose-limiting toxicity was above a threshold.

Hypothetical data scenarios were chosen to reflect potential on-study constellations and the model’s recommended escalation decisions were considered. Complete trials were simulated to demonstrate that the model gave reasonable MTD recommendations. As template programmes were used, simulating 3000 trials took just 4–5 h.

To show the team what they could expect in steering committee meetings, Figure 2 and Table 3 were created before the trial started. They represent an example output for a dose-escalation meeting after one hypothetical patient has taken 5 mg without toxicity. The Bayesian model considered doses up to 10 mg safe as they had low probabilities of overtoxicity (Figure 2, top). It classified the dose level most likely to reach the target toxicity range 16–32%, 40 mg, as too risky for overtoxicity. It recommended testing 10 mg next.

The statisticians explained that the model gives recommendations that the steering committee members can overrule, not binding rules. *The statistical report will clearly describe the dose-escalation decisions that were made in the trial.*
