# Supplementary material for: Embracing model-based designs for dose-finding trials
Source: Br J Cancer. 2017 Jun 29;117(3):332–9. doi: 10.1038/bjc.2017.186 (PMC5537496; doi:10.1038/bjc.2017.186)
Supplement: Supplementary Material [file bjc2017186x1.docx]

**Supplementary material**

**Supplementary table A**

| Pubmed search strategy for Continual Reassessment Method and 3+3   1. "Continual Reassessment Method" [tiab] OR "Continual Reassessment Methods" [tiab] (204) 2. "continual reassessment" [tiab] OR "continuous reassessment" [tiab] (274)   3. CRM [tiab] (2517)  4. TITE-CRM [tiab] (15)  5. mCRM [tiab] (12)  6. B-CRM [tiab] (3)  7. #1 OR #2 OR #3 OR #4 OR #5 OR #6 (2694)  8. "3 + 3" [tiab] (118334)  9. "A + B design" [tiab] OR "A + B designs" [tiab] (65)  10. "rolling six" [tiab] OR "rolling-6" [tiab] OR "rolling 6" [tiab] OR "rolling-six" [tiab] (22)  11. "algorithm based" [tiab] OR "algorithm-based" [tiab] (4553)  12. "rule based" [tiab] OR "rule-based" [tiab] (2380)  13. "adaptive design" [tiab] OR "adaptive designs" [tiab] (483)  14. "traditional escalation rule" [tiab] (0)  15. "standard design" OR "standard designs" [tiab] (220)  16. "algorithmic design" OR "algorithmic designs" [tiab] (23)  17. #8 OR #9 OR #10 OR #11 OR #12 OR #13 OR #14 OR #15 OR #16 (126009)  18. #7 AND #17 (61)  19. Clinical Trials, Phase I as Topic [mh] OR Clinical Trial, Phase I [pt] (18782)  20. "phase I" [tiab] OR "phase one" [tiab] OR "phase 1" [tiab] OR "first-in-man" [tiab] OR "first-in-human" [tiab] OR "first in man" [tiab] OR "first in human" [tiab] OR "first-in-person" [tiab] OR "first in person" [tiab] OR “initial human” [tiab] OR "dose-finding trial" [tiab] OR "dose-finding trials" [tiab] OR "dose-finding study" [tiab] OR "dose-finding studies" [tiab] OR "dose-finding design" [tiab] OR "dose escalation trial" [tiab] OR "dose escalation trials" [tiab] OR "dose escalation study" [tiab] OR "dose escalation studies" [tiab] OR "dose escalation design" [tiab] OR "dose-ranging clinical trial" [tiab] OR "dose-ranging clinical trials" [tiab] OR "dose-ranging trial" [tiab] OR "dose-ranging trials" [tiab] (46574)  21. #19 OR #20 (51905)  22. #18 AND #21 (38) |
| --- |

**Supplementary table B**

| Embase search strategy for Continual Reassessment Method and 3+3  1. Continual Reassessment Method/ (37)  2. (continual adj reassessment adj method$).ti,ab. (321)  3. ((continual OR continuous) adj reassessment).ti,ab. (425)  4. Quasi-CRM.ti,ab. (4)  5. CRM.ti,ab. (3403)  6. TITE-CRM.ti,ab. (30)  7. mCRM.ti,ab. (15)  8. B-CRM.ti,ab. (4)  9. 1 OR 2 OR 3 OR 4 OR 5 OR 6 OR 7 OR 8 (3688)  10. "3+3".ti,ab. (45474)  11. "3 plus 3".ti,ab. (13)  12. "A+B design$".ti,ab. (80)  13. "rolling six".ti,ab. (24)  14. "rolling-six".ti,ab. (24)  15. "rolling 6".ti,ab. (28)  16. "rolling-6".ti,ab. (28)  17. (algorithm adj based).ti,ab. (5850)  18. (algorithm-based).ti,ab. (5850)  19. "rule based".ti,ab. (2683)  20. "rule-based".ti,ab. (2683)  21. (adaptive adj design$).ti,ab. (720)  22. (traditional adj escalation adj rule).ti,ab. (1)  23. (standard adj design$).ti,ab. (416)  24. (algorithmic adj design$).ti,ab. (20)  25. (empirically adj based adj traditional adj method$).ti,ab. (1)  26. (empirically-based adj traditional adj method$).ti,ab. (1)  27. 10 OR 11 OR 12 OR 13 OR 14 OR 15 OR 16 OR 17 OR 18 OR 19 OR 20 OR 21 OR 22 OR 23 OR 24 OR 25 OR 26 (55237)  28. 9 AND 27 (80)  29. "Phase 1 clinical trial (topic)"/ (9214)  30. Phase I Clinical Trial/ (29437)  31. "phase I".ti,ab. (47733)  32. "phase 1".ti,ab. (15020)  33. "phase one".ti,ab. (1459)  34. "first-in-man".ti,ab. (1169)  35. "first in man".ti,ab. (1169)  36. "first-in-human".ti,ab. (1654)  37. "first in human".ti,ab. (1654)  38. "first-in-person".ti,ab. (8)  39. "first in person".ti,ab. (8)  40. (initial adj human).ti,ab. (344)  41. "dose-finding trial$".ti,ab. (301)  42. "dose-finding stud$".ti,ab. (2024)  43. "dose-finding design$".ti,ab. (82)  44. "dose escalation trial$".ti,ab. (1051)  45. "dose escalation stud$".ti,ab. (3333)  46. "dose escalation design$".ti,ab. (486)  47. "dose-ranging clinical trial$".ti,ab. (33)  48. "dose-ranging trial$".ti,ab. (254)  49. Drug Dose Escalation/ (16490)  50. Radiation Dose Escalation/ (386)  51. 29 OR 30 OR 31 OR 32 OR 33 OR 34 OR 35 OR 36 OR 37 OR 38 OR 39 OR 40 OR 41 OR 42 OR 43 OR 44 OR 45 OR 46 OR 47 OR 48 OR 49 OR 50 (95153)  52. 28 AND 51 (66) |
| --- |

**Supplementary Table C: Barriers to embracing model based design questionnaire**

| **Number** | **Question** |  | **Response options** |  |  |  |  |  |
| --- | --- | --- | --- | --- | --- | --- | --- | --- |
| Q1_all | Are you: |  | Chief Investigator | Statistician | Trial Manager | Funder | Other | Please specify |
| Q2 | How long have you worked with dose finding studies? |  | I have never worked with dose finding studies | 0-2 years | 3-5 years | 6-10 years | 11-20 years | 20+ years |
| Q3 | Have you ever been involved in a dose finding study that, rather than using 3+3 or another rule-based design, used an alternative? |  | yes | no | don't know |  |  |  |
| Q4_Stats | Do you have access to software to support alternative approaches to 3+3 and other rule-based designs? |  | yes | no | don't know |  |  |  |
| Q4_others | Is appropriate statistical support available to you to undertake alternative approaches to 3+3 and other rule-based designs? |  | yes | no | don't know |  |  |  |
| Q5_Stats | When designing a trial, how often do you consider alternatives to 3+3 and rule-based designs |  | always | often | not very often | never | don't know |  |
| Q5_others | When designing a trial, how often is there discussion about alternative designs to the 3+3 or other rule-based designs? |  | always | often | not very often | never | don't know |  |
| Q6 | In your experience, how often is the following a barrier to using alternative approaches to 3+3 and other rule-based designs ? | CI prefers 3 + 3 design | always | often | not very often | never | don't know |  |
|  |  | Statistician prefers 3 + 3 design | always | often | not very often | never | don't know |  |
|  |  | Funder prefers 3 + 3 design | always | often | not very often | never | don't know |  |
|  |  | Journal prefers 3 + 3 design | always | often | not very often | never | don't know |  |
|  |  | Regulator prefers 3 + 3 design | always | often | not very often | never | don't know |  |
| Q7 | In your experience, how often is the following a barrier to using alternative approaches to 3+3 and other rule-based designs ? | Statisticians’ lack of knowledge about alternatives to 3+3 - | always | often | not very often | never | don't know |  |
|  |  | CIs’ lack of knowledge about alternatives to 3+3 | always | often | not very often | never | don't know |  |
|  |  | Regulators’ lack of knowledge about alternatives to 3+3 - Always | always | often | not very often | never | don't know |  |
|  |  | Funders’ lack of knowledge about alternatives to 3+3 - Always | always | often | not very often | never | don't know |  |
|  |  | Trial Managers' lack of knowledge about alternatives to 3+3 - Always | always | often | not very often | never | don't know |  |
| Q8 | In my experience the following is a barrier to using alternative approaches to 3+3 and other rule-based designs: | Lack of suitable training - Strongly agree | strongly agree | agree | disagree | strongly disagree | don't know |  |
|  |  | Lack of time to attend training - Strongly agree | strongly agree | agree | disagree | strongly disagree | don't know |  |
|  |  | Lack of time to study what I learnt about alternative approaches - Strongly agree | strongly agree | agree | disagree | strongly disagree | don't know |  |
|  |  | Lack of opportunities to apply what I learnt - Strongly agree | strongly agree | agree | disagree | strongly disagree | don't know |  |
| Q9 | In my experience, the requirement to obtain quick, reliable data to inform adaptation forms a particular barrier to using alternatives to 3+3 and other rule-based designs? |  | strongly agree | agree | disagree | strongly disagree | don't know |  |
| Q10 | In my experience, the lack of consistency in the literature supporting alternatives to 3+3 and other rule-based designs is a barrier to using them |  | strongly agree | agree | disagree | strongly disagree | don't know |  |
| Q11 | In my experience, the limited resources available to design a study prior to funding constrain our ability to use alternatives to 3+3 and other rule-based designs |  | strongly agree | agree | disagree | strongly disagree | don't know |  |
| Q12 | In my experience, funders do not respond positively to the increased costs involved in the implementation of designs that are more complex than 3+3 and other rule-based designs |  | strongly agree | agree | disagree | strongly disagree | don't know |  |
| Q13 | In my experience, the short turnaround for designing studies is a barrier to considering alternatives to 3+3 and other rule-based designs? |  | strongly agree | agree | disagree | strongly disagree | don't know |  |
| Q14 | I previously had a poor experience of using an alternative approach to 3+3/rule-based designs |  | Yes | No | Please provide brief details |  |  |  |
| Q15 | Do you have any other concerns about using alternative approaches to 3+3/rule-based designs? |  | Yes | No | Please specify |  |  |  |

**Supplementary Table D: Free text response to Q14 and Q15**

| Two prior bad experiences: 1) Bayesian method which estimated the next dose level - we opened several dose cohorts over several months all around the MTD when this could have been determined faster by 3+3 ii) CRM method where some of the participating sites did not turnaround eCRF data entry in a timely manner e.g. 21 days instead of 5 which delayed dose decisions. |
| --- |
| CRM / Bayesian methods require real time data capture which may not be resourced in an academic centre, real time PK analysis and real time statistical support |
| We are often looking for data (whether tolerability or pharmacodynamic) at multiple does levels and whilst model-based approaches fit a curve (and estimate optimal dose most accurately), the information at others doses can be limited to demonstrate a dose-effect or investigate exploratory biomarkers that may give a better readout of optimal dose. We can then recruit more patients at those other levels but then lose some of the efficiency gains. |
| As a phase I trialist I have used a variety of designs, 3+3 has the advantage that it is reliable and works |
| Not yet convinced they provide more reliable dose finding |
| I have only experience of one true alternative trial design in phase I and it actually worked well but (1) I don’t think it saved time or reduced the number of cohorts studied (2) on one occasion at least the investigators and study staff over-ruled the next dose level, feeling it was too high and not safe enough (3) it as very complex (only one person seemed to know what the next dose level would be for a patient) |
| I would like to use more efficient approaches more frequently. However, with FIM studies of novel agents in oncology, nowadays, there are often too many unknowns to make ambitious dose escalation schemes acceptable (eg. PK, PD and toxicities not predictable). The other main barrier is lack of availability of good statisticians to advise on design |
| have had good experiences using rolling 6, CRM and other adaptive designs |
| main issue is 'selling' them to some funders and fellow clinicians |
| the trial took much, much longer and far too many patients were recruited into the lower doses compared to a 3+3 |
| Lack of dedicated statistical support and experience of e.g. Bayesian designs that led to slow trials and far too many patients for a phase 1 |
| In various discussions with other CIs etc., there seems to be a consensus that there is no current design which is consistently superior to 3+3 |
| Although I have never used an alternative approach I am currently in the process of incorporating 'alternative' elements into a phase I design so have awareness of the issues. |
| Much slower and binary based on DLTs with too little flexibility to use other toxicity data, PD and PK data |
| To what extend the rule-based designs can be accepted by the PI? How to standardize procedure of rule-based designs? |
| Need to be confident enough to design, analyse, write up, persuade CI, and communicate all of that with non-statistical audiences - easier if working in an environment where others are doing this regularly, for support - but accessible training materials / software / literature would help |
| Not involved in such studies, but if required to be I would want a generally-accepted theoretical approach with easily accessible software (much more of an issue for those who don't normally do these studies than for experts). The 3+3 design meets these requirements |
